# Benzyl *N*-[2-(1*H*-indol-3-yl)eth­yl]dithio­carbamate

**DOI:** 10.1107/S1600536807066524

**Published:** 2007-12-18

**Authors:** Nagarajan Vembu, Frank R. Fronczek, Celine Petit, Marc Devocelle

**Affiliations:** a Department of Chemistry, Urumu Dhanalakshmi College, Tiruchirappalli 620 019, India; b Department of Chemistry, Louisiana State University, Baton Rouge, LA 70803-1804, USA; c Centre for Synthesis and Chemical Biology, Department of Pharmaceutical and Medicinal Chemistry, Royal College of Surgeons in Ireland, 123 St Stephen’s Green, Dublin 2, Ireland

## Abstract

The indole and phenyl ring systems in the title compound, C_18_H_18_N_2_S_2_, are nearly coplanar, the indole and phenyl planes forming a dihedral angle of 6.5 (1)°. Supra­molecular aggregation is effected by N—H⋯S, C—H⋯S, N—H⋯π and C—H⋯π inter­actions. The crystal studied exhibited inversion twinning.

## Related literature

For a detailed account of the indole­amine 2,3-dioxy­genase (IDO) inhibitory properties of the title compound and other brassinin derivatives, see: Gaspari *et al.* (2006 ▶) and references cited therein. For hydrogen-bond criteria, see: Desiraju & Steiner (1999 ▶); Desiraju (1989 ▶). For graph-set notations, see: Bernstein *et al.* (1995 ▶); Etter (1990 ▶).
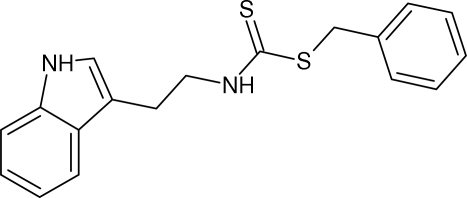


## Experimental

### 

#### Crystal data

C_18_H_18_N_2_S_2_
*M* *_r_* = 326.46
Monoclinic, 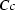

*a* = 34.554 (10) Å
*b* = 5.459 (2) Å
*c* = 8.875 (3) Å
β = 102.522 (18)°
*V* = 1634.3 (9) Å^3^
*Z* = 4
Mo *K*α radiation
μ = 0.32 mm^−1^
*T* = 90 K
0.30 × 0.27 × 0.05 mm

#### Data collection

Nonius KappaCCD diffractometer with an Oxford Cryosystems Cryostream cooler
Absorption correction: multi-scan (*SCALEPACK*; Otwinowski & Minor, 1997 ▶) *T*_min_ = 0.909, *T*_max_ = 0.984
14452 measured reflections
3638 independent reflections
2846 reflections with *I* > 2σ(*I*)
*R*_int_ = 0.013

#### Refinement

*R*[*F* ^2^ > 2σ(*F* ^2^)] = 0.038
*wR*(*F* ^2^) = 0.083
*S* = 1.06
3638 reflections
271 parameters
2 restraints
All H-atom parameters refined
Δρ_max_ = 0.28 e Å^−3^
Δρ_min_ = −0.36 e Å^−3^
Absolute structure: Flack (1983 ▶), 1615 Friedel pairs
Flack parameter: 0.44 (6)

### 

Data collection: *COLLECT* (Nonius, 2000 ▶); cell refinement: *DENZO* and *SCALEPACK* (Otwinowski & Minor, 1997 ▶); data reduction: *DENZO* and *SCALEPACK*; program(s) used to solve structure: *SHELXS97* (Sheldrick, 1997 ▶); program(s) used to refine structure: *SHELXL97* (Sheldrick, 1997 ▶); molecular graphics: *PLATON* (Spek, 2003 ▶); software used to prepare material for publication: *SHELXL97*.

## Supplementary Material

Crystal structure: contains datablocks I, global. DOI: 10.1107/S1600536807066524/sj2455sup1.cif

Structure factors: contains datablocks I. DOI: 10.1107/S1600536807066524/sj2455Isup2.hkl

Additional supplementary materials:  crystallographic information; 3D view; checkCIF report

## Figures and Tables

**Table 1:** Hydrogen-bond geometry (Å, °) *Cg*1 is the centroid of the N1/C2–C5 ring, *Cg*2 that of the C4–C9 ring and *Cg*3 that of the C17–C22 ring.

*D*—H⋯*A*	*D*—H	H⋯*A*	*D*⋯*A*	*D*—H⋯*A*
C11—H11*B*⋯S14	0.95 (3)	2.65 (2)	3.103 (2)	109.6 (17)
C16—H16*A*⋯S14	1.00 (2)	2.80 (2)	3.222 (2)	106.1 (15)
N12—H12⋯S14^i^	0.84 (3)	2.50 (3)	3.283 (2)	156 (2)
C16—H16*A*⋯S15^ii^	1.00 (2)	2.78 (2)	3.642 (3)	144.8 (17)
N1—H1⋯*Cg*2^iii^	0.84 (3)	2.658	3.373	143.65
C8—H8⋯*Cg*2^ii^	0.95 (3)	3.244	3.868	124.79
C9—H9⋯*Cg*1^ii^	0.94 (3)	2.807	3.574	140.03
C18—H18⋯*Cg*3^iv^	1.04 (3)	3.174	4.053	142.60
C21—H21⋯*Cg*3^v^	0.93 (3)	3.212	3.946	136.93

Symmetry codes: (i) 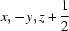; (ii) 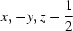; (iii) 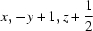; (iv) 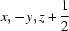; (v) 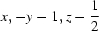.

**Fig. 1.**
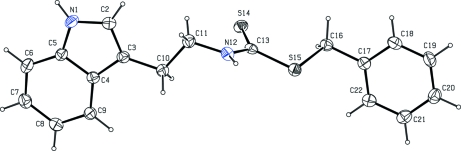
The asymmetric unit of (I) with the atoms labelled and displacement ellipsoids depicted at the 50% probability level for all non-H atoms. H-atoms are drawn as spheres of arbitrary radius.

**Fig. 2.**
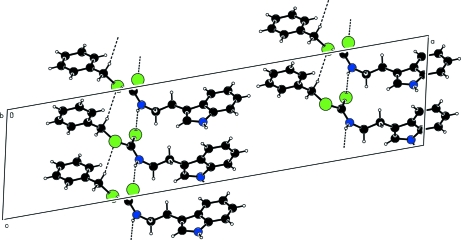
The molecular packing viewed down the b-axis. Dashed lines represent the weak N—H···S and C—H···S interactions within the lattice.

## supplementary crystallographic information

### Comment

The enzyme indoleamine 2,3-dioxygenase (IDO) has been reported to play a role in tumour immunosuppression. IDO inhibitors have been reported to be novel therapeutics for cancer treatment. The natural product brassinin has been shown to be a moderately active competitive IDO inhibitor. The title compound, (I), Fig. 1, is a brassinin derivative and its IDO inhibitory properties have been reported (Gaspari *et al.*, 2006). The present investigation is aimed at the study of the molecular and supramolecular architecture of the title compound. This study may serve as a forerunner to an investigation of the correlation between the molecular and supramolecular features of this compound with its biological activity.

In (I), the dithiocarbamate moiety is essentially planar, as shown by the small deviation of N12 [0.0036 (6) Å], C13 [-0.009 (2) Å], S14 [0.0032 (5) Å] & S15 [0.0027 (5) Å] atoms from their mean plane. The interplanar angle between the indole and the phenyl ring is 6.5 (1)° thereby confirming their near coplanarity.

The crystal structure of (I) is stabilized by the interplay of N—H···S, C—H···S, N—H···π and C—H···π interactions, Fig. 2, Table 1. The H-bond distances found in (I) agree with those reported in literature (Desiraju & Steiner, 1999; Desiraju, 1989). The C11—H11B···S14 interaction generates a motif of graph set (Bernstein *et al.*, 1995; Etter, 1990) S(5). Another S(5) motif is formed by the C16—H16···S14 interaction. The N12—H12···S14^i^ interaction generates an infinite one-dimensional chain along [001]. The N12—H12···S14^i^ and C16—H16A···S15^ii^ interactions generate a binary motif of graph set *R*^2^_2_(9). The C8—H8···*Cg*2^ii^ and C9—H9···*Cg*1^ii^ interactions generate an *R*^2^_2_(6) motif in which each of the aromatic rings are considered as single acceptor atoms. *Cg*1 is the centroid of the N1, C2, C3, C4 & C5 ring, *Cg*2 that of the C4, C5, C6, C7, C8 & C9 ring and *Cg*3 that of the C17, C18, C19, C20, C21 & C22 ring, Table 1.

### Experimental

The title compound was prepared by the reported procedure (Gaspari *et al.*, 2006). Diffraction quality crystals were obtained by recrystallizing the crude product from a 1:1 mixture of dichloromethane and petroleum ether.

### Refinement

All H-atoms were located in difference maps and their positions and isotropic displacement parameters freely refined. Refinement of the Flack (1983) parameter indicated an inversion twin with components of slightly different size.

### Crystal data

**Table:**

C_18_H_18_N_2_S_2_	*F*_000_ = 688
*M**_r_* = 326.46	*D*_x_ = 1.327 Mg m^−^^3^
Monoclinic, *C**c*	Mo *K*α radiation λ = 0.71073 Å
Hall symbol: C -2yc	Cell parameters from 1986 reflections
*a* = 34.554 (10) Å	θ = 2.5–28.3º
*b* = 5.459 (2) Å	µ = 0.32 mm^−^^1^
*c* = 8.875 (3) Å	*T* = 90 K
β = 102.522 (18)º	Plate, colorless
*V* = 1634.3 (9) Å^3^	0.30 × 0.27 × 0.05 mm
*Z* = 4	

### Data collection

**Table:**

Nonius KappaCCD diffractometer with an Oxford Cryosystems Cryostream cooler	3638 independent reflections
Radiation source: fine-focus sealed tube	2846 reflections with *I* > 2σ(*I*)
Monochromator: graphite	*R*_int_ = 0.013
*T* = 90 K	θ_max_ = 28.3º
ω scans with κ offsets	θ_min_ = 3.6º
Absorption correction: multi-scan(SCALEPACK; Otwinowski & Minor, 1997)	*h* = −45→45
*T*_min_ = 0.909, *T*_max_ = 0.984	*k* = −7→7
14452 measured reflections	*l* = −11→11

### Refinement

**Table:**

Refinement on *F*^2^	Hydrogen site location: inferred from neighbouring sites
Least-squares matrix: full	All H-atom parameters refined
*R*[*F*^2^ > 2σ(*F*^2^)] = 0.038	*w* = 1/[σ^2^(*F*_o_^2^) + (0.0459*P*)^2^ + 0.298*P*] where *P* = (*F*_o_^2^ + 2*F*_c_^2^)/3
*wR*(*F*^2^) = 0.083	(Δ/σ)_max_ < 0.001
*S* = 1.06	Δρ_max_ = 0.28 e Å^−^^3^
3638 reflections	Δρ_min_ = −0.36 e Å^−^^3^
271 parameters	Extinction correction: none
2 restraints	Absolute structure: Flack (1983), 1615 Friedel pairs
Primary atom site location: structure-invariant direct methods	Flack parameter: 0.44 (6)
Secondary atom site location: difference Fourier map	

### Special details

**Table:**

Geometry. All e.s.d.'s (except the e.s.d. in the dihedral angle between two l.s. planes) are estimated using the full covariance matrix. The cell e.s.d.'s are taken into account individually in the estimation of e.s.d.'s in distances, angles and torsion angles; correlations between e.s.d.'s in cell parameters are only used when they are defined by crystal symmetry. An approximate (isotropic) treatment of cell e.s.d.'s is used for estimating e.s.d.'s involving l.s. planes.
Refinement. Refinement of *F*^2^ against ALL reflections. The weighted *R*-factor *wR* and goodness of fit *S* are based on *F*^2^, conventional *R*-factors *R* are based on *F*, with *F* set to zero for negative *F*^2^. The threshold expression of *F*^2^ > σ(*F*^2^) is used only for calculating *R*-factors(gt) *etc*. and is not relevant to the choice of reflections for refinement. *R*-factors based on *F*^2^ are statistically about twice as large as those based on *F*, and *R*- factors based on ALL data will be even larger.

### Fractional atomic coordinates and isotropic or equivalent isotropic displacement parameters (Å^2^)

**Table:**

	*x*	*y*	*z*	*U*_iso_*/*U*_eq_	
N1	0.46964 (6)	−0.5161 (4)	1.1785 (2)	0.0254 (4)	
C2	0.43138 (7)	−0.4259 (4)	1.1409 (3)	0.0226 (5)	
C3	0.42960 (6)	−0.2308 (4)	1.0432 (2)	0.0213 (4)	
C4	0.46901 (6)	−0.1951 (4)	1.0199 (2)	0.0193 (4)	
C5	0.49326 (6)	−0.3788 (4)	1.1043 (2)	0.0212 (5)	
C6	0.53368 (7)	−0.4004 (4)	1.1042 (3)	0.0255 (5)	
C7	0.54953 (7)	−0.2338 (4)	1.0168 (2)	0.0267 (5)	
C8	0.52616 (7)	−0.0488 (4)	0.9323 (3)	0.0251 (5)	
C9	0.48610 (6)	−0.0264 (4)	0.9334 (3)	0.0221 (5)	
C10	0.39402 (6)	−0.0822 (4)	0.9696 (3)	0.0220 (5)	
C11	0.35556 (7)	−0.1763 (4)	1.0067 (3)	0.0223 (5)	
N12	0.32086 (5)	−0.0433 (4)	0.9222 (2)	0.0220 (4)	
C13	0.29978 (6)	−0.1080 (4)	0.7835 (2)	0.0197 (4)	
S14	0.309251 (17)	−0.35477 (9)	0.68619 (5)	0.02339 (14)	
S15	0.261499 (15)	0.10448 (9)	0.71423 (4)	0.02185 (14)	
C16	0.22633 (7)	−0.0708 (4)	0.5720 (3)	0.0213 (5)	
C17	0.19391 (6)	0.1051 (4)	0.4973 (2)	0.0201 (5)	
C18	0.15460 (7)	0.0621 (5)	0.5069 (3)	0.0264 (5)	
C19	0.12465 (7)	0.2181 (5)	0.4336 (3)	0.0307 (5)	
C20	0.13319 (8)	0.4170 (4)	0.3499 (3)	0.0288 (5)	
C21	0.17215 (7)	0.4632 (4)	0.3412 (3)	0.0264 (5)	
C22	0.20258 (7)	0.3073 (4)	0.4145 (2)	0.0232 (5)	
H1	0.4773 (8)	−0.640 (5)	1.233 (3)	0.027 (7)*	
H2	0.4095 (7)	−0.498 (4)	1.181 (3)	0.018 (5)*	
H6	0.5501 (7)	−0.527 (5)	1.159 (3)	0.024 (6)*	
H7	0.5796 (8)	−0.242 (5)	1.017 (3)	0.024 (6)*	
H8	0.5382 (8)	0.073 (5)	0.881 (3)	0.040 (8)*	
H9	0.4699 (8)	0.095 (5)	0.878 (3)	0.033 (7)*	
H10A	0.3964 (7)	0.086 (5)	0.999 (3)	0.032 (7)*	
H10B	0.3906 (7)	−0.083 (4)	0.866 (3)	0.024 (6)*	
H11A	0.3577 (7)	−0.155 (4)	1.127 (3)	0.016 (6)*	
H11B	0.3517 (7)	−0.341 (5)	0.973 (3)	0.015 (5)*	
H12	0.3127 (7)	0.076 (5)	0.967 (3)	0.024 (6)*	
H16A	0.2415 (7)	−0.147 (4)	0.500 (3)	0.016 (6)*	
H16B	0.2166 (7)	−0.205 (5)	0.624 (3)	0.026 (6)*	
H18	0.1495 (8)	−0.084 (5)	0.576 (3)	0.033 (7)*	
H19	0.0993 (10)	0.188 (5)	0.433 (3)	0.037 (7)*	
H20	0.1109 (10)	0.512 (6)	0.297 (4)	0.047 (8)*	
H21	0.1775 (7)	0.597 (5)	0.283 (3)	0.028 (7)*	
H22	0.2336 (8)	0.353 (4)	0.412 (3)	0.026 (6)*	

### Atomic displacement parameters (Å^2^)

**Table:**

	*U*^11^	*U*^22^	*U*^33^	*U*^12^	*U*^13^	*U*^23^
N1	0.0281 (10)	0.0213 (10)	0.0267 (10)	0.0045 (8)	0.0061 (8)	0.0075 (9)
C2	0.0258 (12)	0.0206 (11)	0.0221 (11)	−0.0015 (9)	0.0067 (9)	−0.0007 (9)
C3	0.0239 (11)	0.0188 (11)	0.0201 (10)	−0.0019 (8)	0.0023 (8)	−0.0004 (9)
C4	0.0230 (11)	0.0166 (10)	0.0181 (11)	−0.0013 (8)	0.0039 (8)	−0.0030 (9)
C5	0.0230 (12)	0.0199 (11)	0.0194 (11)	0.0015 (8)	0.0015 (9)	0.0000 (8)
C6	0.0271 (12)	0.0231 (12)	0.0238 (12)	0.0058 (9)	−0.0001 (10)	−0.0001 (10)
C7	0.0230 (13)	0.0260 (13)	0.0308 (13)	0.0004 (9)	0.0051 (9)	−0.0044 (10)
C8	0.0247 (12)	0.0238 (12)	0.0267 (12)	−0.0035 (9)	0.0052 (9)	−0.0014 (10)
C9	0.0217 (12)	0.0209 (11)	0.0219 (11)	−0.0005 (9)	0.0009 (9)	−0.0005 (9)
C10	0.0221 (12)	0.0214 (12)	0.0216 (13)	−0.0001 (8)	0.0027 (9)	−0.0003 (10)
C11	0.0234 (12)	0.0223 (12)	0.0204 (12)	−0.0001 (9)	0.0031 (9)	−0.0006 (9)
N12	0.0216 (10)	0.0238 (10)	0.0215 (10)	−0.0003 (8)	0.0069 (8)	−0.0037 (8)
C13	0.0199 (11)	0.0208 (11)	0.0194 (11)	−0.0050 (8)	0.0069 (8)	0.0015 (9)
S14	0.0290 (3)	0.0189 (3)	0.0217 (3)	0.0002 (2)	0.0043 (2)	−0.0010 (2)
S15	0.0224 (3)	0.0210 (3)	0.0213 (3)	−0.0007 (2)	0.0029 (2)	−0.0029 (2)
C16	0.0220 (12)	0.0203 (10)	0.0216 (11)	−0.0019 (9)	0.0046 (9)	−0.0006 (10)
C17	0.0227 (11)	0.0197 (11)	0.0174 (10)	−0.0015 (8)	0.0031 (8)	−0.0042 (9)
C18	0.0244 (12)	0.0252 (12)	0.0305 (12)	−0.0041 (9)	0.0081 (9)	−0.0020 (10)
C19	0.0183 (12)	0.0314 (13)	0.0422 (14)	−0.0011 (10)	0.0058 (10)	−0.0043 (11)
C20	0.0291 (13)	0.0241 (12)	0.0304 (13)	0.0045 (10)	0.0008 (10)	−0.0028 (11)
C21	0.0345 (14)	0.0211 (11)	0.0229 (12)	0.0015 (10)	0.0046 (9)	−0.0015 (10)
C22	0.0276 (12)	0.0220 (11)	0.0202 (10)	−0.0018 (9)	0.0057 (9)	−0.0020 (9)

### Geometric parameters (Å, °)

**Table:**

N1—C5	1.377 (3)	C11—H11A	1.06 (2)
N1—C2	1.382 (3)	C11—H11B	0.95 (3)
N1—H1	0.84 (3)	N12—C13	1.335 (3)
C2—C3	1.366 (3)	N12—H12	0.84 (3)
C2—H2	0.99 (3)	C13—S14	1.670 (2)
C3—C4	1.434 (3)	C13—S15	1.767 (2)
C3—C10	1.499 (3)	S15—C16	1.822 (2)
C4—C9	1.408 (3)	C16—C17	1.515 (3)
C4—C5	1.413 (3)	C16—H16A	1.00 (2)
C5—C6	1.402 (3)	C16—H16B	0.97 (3)
C6—C7	1.383 (3)	C17—C22	1.394 (3)
C6—H6	0.96 (3)	C17—C18	1.399 (3)
C7—C8	1.405 (3)	C18—C19	1.388 (4)
C7—H7	1.04 (3)	C18—H18	1.04 (3)
C8—C9	1.391 (3)	C19—C20	1.383 (4)
C8—H8	0.95 (3)	C19—H19	0.89 (3)
C9—H9	0.94 (3)	C20—C21	1.388 (4)
C10—C11	1.526 (3)	C20—H20	0.96 (3)
C10—H10A	0.95 (3)	C21—C22	1.399 (3)
C10—H10B	0.90 (3)	C21—H21	0.93 (3)
C11—N12	1.462 (3)	C22—H22	1.10 (3)
			
C5—N1—C2	109.01 (19)	C10—C11—H11A	107.8 (12)
C5—N1—H1	124.7 (18)	N12—C11—H11B	105.6 (14)
C2—N1—H1	126.2 (18)	C10—C11—H11B	108.8 (14)
C3—C2—N1	109.9 (2)	H11A—C11—H11B	113.1 (19)
C3—C2—H2	127.5 (13)	C13—N12—C11	124.4 (2)
N1—C2—H2	122.6 (13)	C13—N12—H12	117.8 (17)
C2—C3—C4	106.49 (19)	C11—N12—H12	117.6 (17)
C2—C3—C10	128.1 (2)	N12—C13—S14	124.15 (17)
C4—C3—C10	125.35 (19)	N12—C13—S15	111.41 (16)
C9—C4—C5	118.86 (19)	S14—C13—S15	124.42 (13)
C9—C4—C3	133.7 (2)	C13—S15—C16	103.47 (10)
C5—C4—C3	107.44 (18)	C17—C16—S15	106.81 (15)
N1—C5—C6	130.44 (19)	C17—C16—H16A	115.0 (13)
N1—C5—C4	107.16 (18)	S15—C16—H16A	107.4 (13)
C6—C5—C4	122.40 (19)	C17—C16—H16B	112.9 (14)
C7—C6—C5	117.4 (2)	S15—C16—H16B	108.4 (14)
C7—C6—H6	119.8 (14)	H16A—C16—H16B	106.1 (19)
C5—C6—H6	122.7 (14)	C22—C17—C18	119.2 (2)
C6—C7—C8	121.4 (2)	C22—C17—C16	120.64 (18)
C6—C7—H7	119.0 (14)	C18—C17—C16	120.16 (19)
C8—C7—H7	119.5 (14)	C19—C18—C17	120.2 (2)
C9—C8—C7	121.1 (2)	C19—C18—H18	122.7 (15)
C9—C8—H8	118.6 (17)	C17—C18—H18	117.0 (15)
C7—C8—H8	120.0 (17)	C20—C19—C18	120.6 (2)
C8—C9—C4	118.8 (2)	C20—C19—H19	117.8 (17)
C8—C9—H9	122.9 (17)	C18—C19—H19	121.5 (17)
C4—C9—H9	118.3 (17)	C19—C20—C21	119.7 (2)
C3—C10—C11	113.23 (19)	C19—C20—H20	116.5 (19)
C3—C10—H10A	112.7 (16)	C21—C20—H20	123.8 (19)
C11—C10—H10A	107.0 (16)	C20—C21—C22	120.3 (2)
C3—C10—H10B	110.8 (16)	C20—C21—H21	118.9 (16)
C11—C10—H10B	107.0 (16)	C22—C21—H21	120.8 (16)
H10A—C10—H10B	106 (2)	C17—C22—C21	120.1 (2)
N12—C11—C10	112.22 (19)	C17—C22—H22	120.5 (13)
N12—C11—H11A	109.5 (13)	C21—C22—H22	119.3 (13)
			
C5—N1—C2—C3	−0.1 (2)	C2—C3—C10—C11	−3.0 (3)
N1—C2—C3—C4	−0.7 (2)	C4—C3—C10—C11	175.5 (2)
N1—C2—C3—C10	178.0 (2)	C3—C10—C11—N12	−174.57 (19)
C2—C3—C4—C9	−179.8 (2)	C10—C11—N12—C13	90.3 (2)
C10—C3—C4—C9	1.4 (4)	C11—N12—C13—S14	1.2 (3)
C2—C3—C4—C5	1.3 (2)	C11—N12—C13—S15	−177.21 (16)
C10—C3—C4—C5	−177.5 (2)	N12—C13—S15—C16	−158.35 (15)
C2—N1—C5—C6	−179.1 (2)	S14—C13—S15—C16	23.20 (17)
C2—N1—C5—C4	0.9 (2)	C13—S15—C16—C17	−175.57 (14)
C9—C4—C5—N1	179.51 (19)	S15—C16—C17—C22	62.1 (2)
C3—C4—C5—N1	−1.4 (2)	S15—C16—C17—C18	−119.77 (19)
C9—C4—C5—C6	−0.5 (3)	C22—C17—C18—C19	0.6 (3)
C3—C4—C5—C6	178.6 (2)	C16—C17—C18—C19	−177.6 (2)
N1—C5—C6—C7	179.8 (2)	C17—C18—C19—C20	0.2 (3)
C4—C5—C6—C7	−0.2 (3)	C18—C19—C20—C21	−1.0 (4)
C5—C6—C7—C8	0.5 (3)	C19—C20—C21—C22	1.0 (3)
C6—C7—C8—C9	0.0 (3)	C18—C17—C22—C21	−0.5 (3)
C7—C8—C9—C4	−0.7 (3)	C16—C17—C22—C21	177.59 (19)
C5—C4—C9—C8	0.9 (3)	C20—C21—C22—C17	−0.2 (3)
C3—C4—C9—C8	−177.9 (2)		

### Hydrogen-bond geometry (Å, °)

**Table:**

*D*—H···*A*	*D*—H	H···*A*	*D*···*A*	*D*—H···*A*
C11—H11B···S14	0.95 (3)	2.65 (2)	3.103 (2)	109.6 (17)
C16—H16A···S14	1.00 (2)	2.80 (2)	3.222 (2)	106.1 (15)
N12—H12···S14^i^	0.84 (3)	2.50 (3)	3.283 (2)	156 (2)
C16—H16A···S15^ii^	1.00 (2)	2.78 (2)	3.642 (3)	144.8 (17)
N1—H1···Cg2^iii^	0.84 (3)	2.658	3.373	143.65
C8—H8···Cg2^ii^	0.95 (3)	3.244	3.868	124.79
C9—H9···Cg1^ii^	0.94 (3)	2.807	3.574	140.03
C18—H18···Cg3^iv^	1.04 (3)	3.174	4.053	142.60
C21—H21···Cg3^v^	0.93 (3)	3.212	3.946	136.93

Symmetry codes: (i) *x*, −*y*, *z*+1/2; (ii) *x*, −*y*, *z*−1/2; (iii) *x*, −*y*+1, *z*+1/2; (iv) *x*, −*y*, *z*+1/2; (v) *x*, −*y*−1, *z*−1/2.
